# Intranasal oxytocin selectively modulates the behavior of rhesus monkeys in an expression matching task

**DOI:** 10.1038/s41598-019-51422-3

**Published:** 2019-10-23

**Authors:** Jessica Taubert, Molly Flessert, Ning Liu, Leslie G. Ungerleider

**Affiliations:** 1 Section on Neurocircuitry, Laboratory of Brain and Cognition, National Institute of Mental Health (NIMH), National Institutes of Health, Bethesda, MD United States; 2 State Key Laboratory of Brain and Cognitive Science, Institute of Biophysics, Chinese Academy of Sciences, Beijing, China

**Keywords:** Amygdala, Social behaviour, Object vision

## Abstract

Although the neuropeptide oxytocin (OT) is thought to regulate prosocial behavior in mammals, there is considerable debate as to how intranasal OT influences primate behavior. The aim of this study was to determine whether intranasal OT has a general anxiolytic effect on the performance of rhesus monkeys tasked with matching face stimuli, or a more selective effect on their behavior towards aversive facial expressions. To this end, we developed an innovative delayed match-to-sample task where the exact same trials could be used to assess either a monkey’s ability to match facial expressions or facial identities. If OT has a general affect on behavior, then performance in both tasks should be altered by the administration of OT. We tested four male rhesus monkeys (*Macaca mulatta*) in both the expression and identity task after the intranasal administration of either OT or saline in a within-subjects design. We found that OT inhalation selectively reduced a selection bias against negatively valenced expressions. Based on the same visual input, performance in the identity task was also unaffected by OT. This dissociation provides evidence that intranasal OT affects primate behavior under very particular circumstances, rather than acting as a general anxiolytic, in a highly translatable nonhuman model, the rhesus monkey.

## Introduction

A large number of studies have shown that the administration of oxytocin (OT) impacts a wide variety of behaviors in mammals including materal care and pair-bonding, functioning in the body as both a hormone and a neuropeptide^1–3^. Its effect on primate social cognition is of general interest because there is an argument that intranasal OT could be used as a possible pharmacotherapy for humans^4,5^. However, although the intranasal administration of OT likely affects primate behavior, there is considerable uncertainty as to the exact nature of those behavioral changes.

While many have argued that OT selectively enhances socio-emotional behavior and the salience of socially-relevant visual stimuli^6–9^, others have asserted that the anxiolytic effects of OT can account for the majority of behavioral findings^10–13^. Evidence against the hypothesis that OT acts on the brain regions that promote typical social behavior is the broad range of different behaviors that OT administration has been reported to impact. In primates, a single intranasal dose of OT has been shown to modulate trust^14^, altruism^15,16^, and empathy towards others^17^, inhibit hunger-driven food intake^18^, reduce aggressiveness^19^, increase eye contact^20,21^, improve social learning^22,23^, decrease social vigilance^24,25^, and heighten feelings of envy^26^. Although the manipulation of a general mechanism responsible for regulating stress or anxiety more broadly^12,13,27^ might be able to explain all of these effects, most of the experiments that have found a link between OT treatment and primate behavior have involved a social context or socially-relevant stimuli. Importantly, the debate centered on the type of behaviors that are influenced by the OT compound needs to be resolved before OT can be developed as an effective pharmacotherapy^28^.

In humans and monkeys, there have been numerous reports that OT inhalation alters viewing behavior towards face stimuli^21,29–33^. In particular, the studies that have demonstrated that OT administration selectively modulates attention towards faces conveying fearful expressions has been used to argue that OT effects are stimulus-driven and social in nature^32,33^ and, thus, the OT signaling pathway promotes specific operations involved in processing social stimuli^7,8,20,34^. However, these studies also suffer from the common criticism; that it would be easier to explain these findings in terms of general changes in the internal state of the subjects, such as reduced anxiety, increased motivation, or improved attention^12,13,35,36^. It has been proven difficult to test the difference between these two theoretical accounts because they largely predict the same outcome: namely, that intranasal OT will have a greater effect on trials with higher emotional valence. Therefore, to resolve the debate surrounding the specificity of the OT effect on primate behavior, the challenge has been to develop a task that can effectively rule out the contribution of general mechanisms.

**Figure 1:**
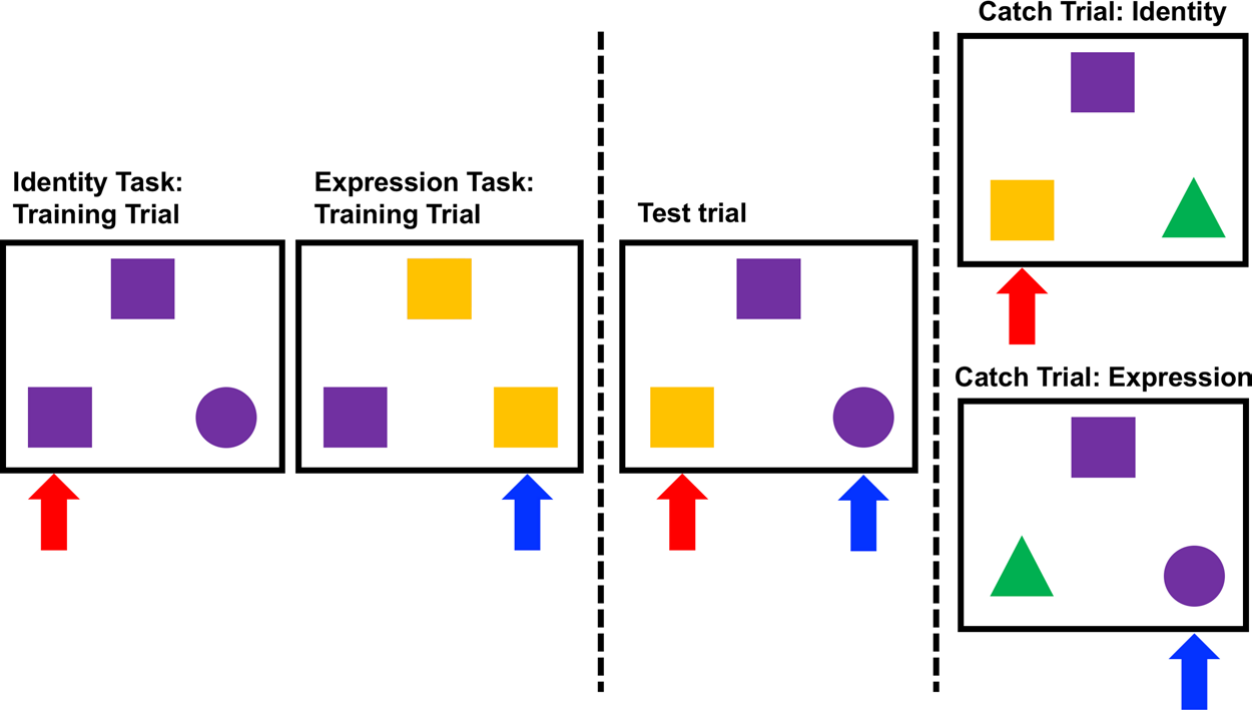
Differences in trial structure across training and test sessions. In these diagrams, face stimuli are represented by colored shapes; identity is represented by shape and expression is represented by color. For illustrative purposes, these diagrams depict the three stimuli (sample, target and distractor) associated with a trial in a white rectangle with a black boarder; the shape representing the sample is on the top, center position with the shapes representing the choices (target and distractor) below. The arrows indicate the target (i.e. the correct choice). The color of the arrow reflects whether the monkeys were matching identity (red) or expression (blue). On the far left are the two training trials (identity and expression, respectively). The monkeys were trained to match identity (shape) while expression (color) was held constant. Likewise, the monkeys were trained to match expression (color) while identity (shape) was held constant. The test trials were the same irrespective of whether the monkeys were matching identity or expression. Nonetheless, these trials were differentially reinforced whereby, if the monkeys matched identity (shape), they had to ignore changes in expression (color). If the monkeys matched expression (color), they had to ignore changes in identity (shape). In Experiment 2, we used “catch trials” to determine whether the monkeys were complying with both matching rules. For example, in the illustrative identity trial, for a monkey tasked with matching identity (shape) but, instead, has been rejecting the matching expression (color) stimulus, the catch trial for the identity task will be impossible because there is no matching expression (color) information to use as a reference, and vice versa in the expression catch trial.

Face perception provides a unique opportunity to test the influence intranasal OT has on primate behavior because, unlike other classes of visual stimuli, at any given time a face will convey multiple independent signals that are thought to be processed by separable neural mechanisms in both humans^37–39^ and monkeys^40^. For example, a face transmits information about who a person is (i.e. their facial identity) and how they are feeling (i.e. their facial expression) simultaneously. This means that, in the present study, we can take advantage of the fact any image of a face will carry both identity and expression cues to develop a new “dual behavior” matching task for testing rhesus monkeys. In the identity matching task, we first trained the subjects to match facial identity in a delayed match-to-sample format (see Fig. 1). Conversely, in the expression matching task, we trained the subjects to match facial expression. Critically, during the test phase of both the identity and expression matching tasks, we presented the exact same trials (identical stimuli in the same configuration). These trials were constructed so that the initial sample could be matched to either a stimulus with the same identity or a stimulus with the same expression (see Fig. 1). Thus, there was no systematic difference between the identity test trials and expression test trials in terms of visual stimulation (the low- and high-level visual attributes were identical), amount of experience (these trials/stimuli were only viewed during the test sessions), or the level of stress they induced. If intranasal OT dampens the subject’s general anxiety or enhances visual processing in a nonspecific way, then the expectation would be that test performance would improve following OT administration, regardless of whether the subject is tasked with matching identity or expression. But, if intranasal OT interacts with a discrete neural circuit responsible for evaluating social salience or emotional valence, then the impact of OT administration should be limited to the expression matching task.

## Results

This study was comprised of two experiments conducted on four male rhesus monkeys. In the main experiment (Experiment 1), we manipulated both the subject’s task (identity or expression matching) and treatment (OT or saline-placebo) in a within-subjects design. In Experiment 2, we repeated Experiment 1 without the manipulation of treatment and added ‘catch trials’ into the test sessions (see Fig. 1). The aim of Experiment 2 was to monitor performance in the catch trials and determine whether the subjects were using identity information to respond accurately in the expression task and vice versa.

**Figure 2:**
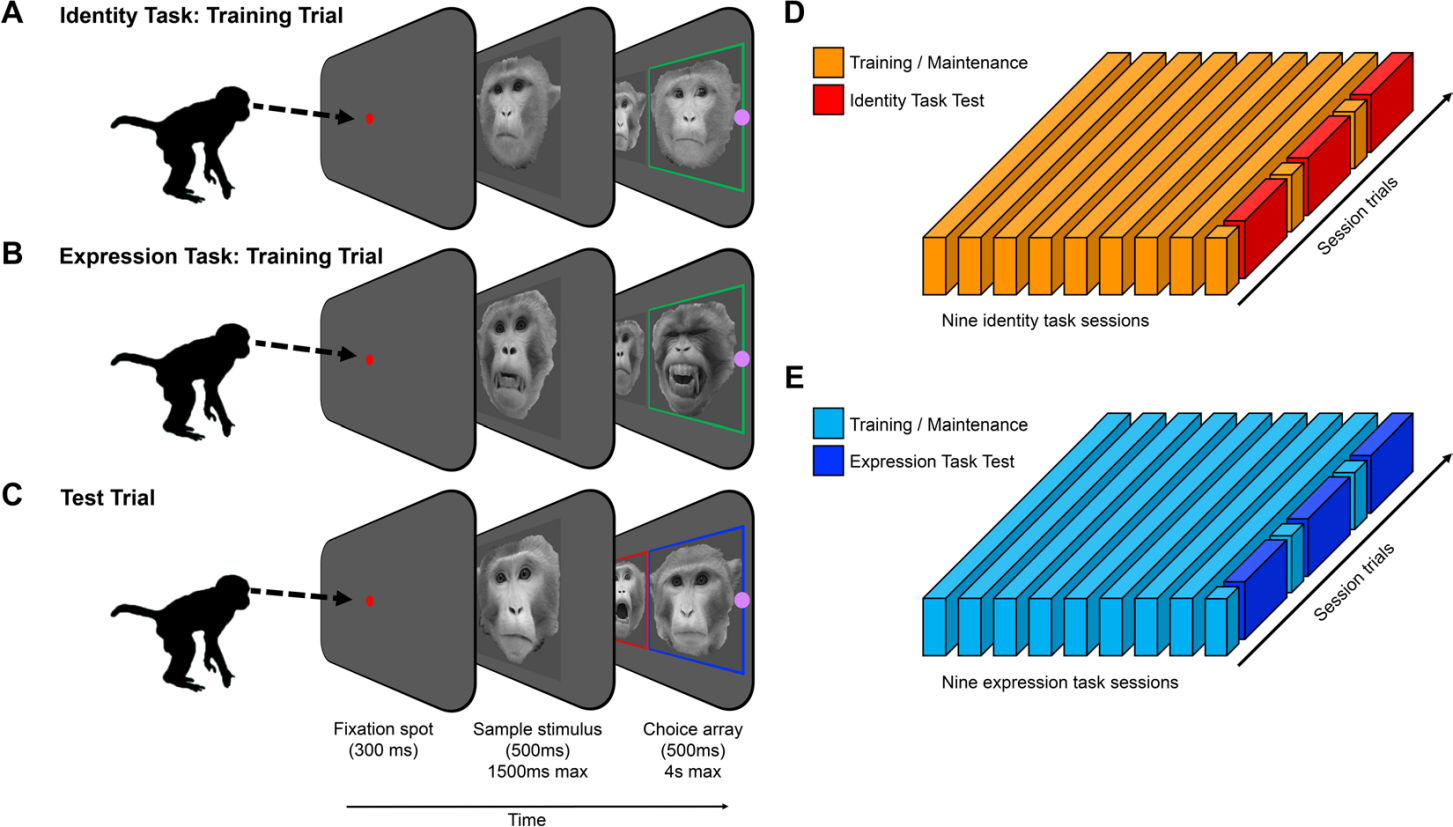
Experiment 1 procedure. (**A)** Illustrative example of an “identity training trial”. The three phases of the trial were the initial fixation period, the presentation of the sample stimulus, and the presentation of the choice array. Here the correct answer is highlighted in green box for illustrative purposes only. **(B)** An example of an “expression training trial”. **(C)** An example of a test trial. The same test trials were presented following identity task training and expression task training; the only difference was which stimulus was reinforced as the correct response. Following identity training, the monkeys were required to recognize identity across a change in expression (red box); following expression training, the monkeys were required to recognize expression across a change in identity (blue box). **(D)** Diagram illustrating the ratio of training/maintenance trials compared to test trials across the identity task sessions. **(E)** Diagram illustrating the ratio of training/maintenance trials compared to test trials across the expression task sessions.

In Experiment 1, the training procedure, which is described in detail in the Materials and Methods section, took eight days (see Fig. 2). Performance in the final training session on the eighth day was analyzed to determine whether the subjects were able to perform both matching tasks. When matching identity, average performance in the final training session was 76.25% (range = 70.18–92.52%). We observed a similar level of performance in the final training session of the expression task (average = 76.49%; range = 70.25–82.69%). We determined that each subject performed both tasks above chance using independent binomial tests (two-tailed, chance = 50%; all *p*-values < 0.0001). Collectively, the training data indicate that all four subjects were proficient at matching the four facial identities and the four facial expressions.

The training procedures for Experiment 2 were identical to those used in Experiment 1 (see Fig. 2A,B). Average performance in the final training session before the identity matching test was 77.81% (range = 71.03–92.9%) with all four subjects performing above chance (two-tailed binomial tests, all *p*-values < 0.0001). Similarly, in the final training session, all subjects were able to match expression at a level above chance (group average = 79.62%; range = 71.75–91.04%; two-tailed binomial tests, all *p*-values < 0.0001).

### Experiment 1: The main effect of intranasal OT on identity and expression matching

**Figure 3:**
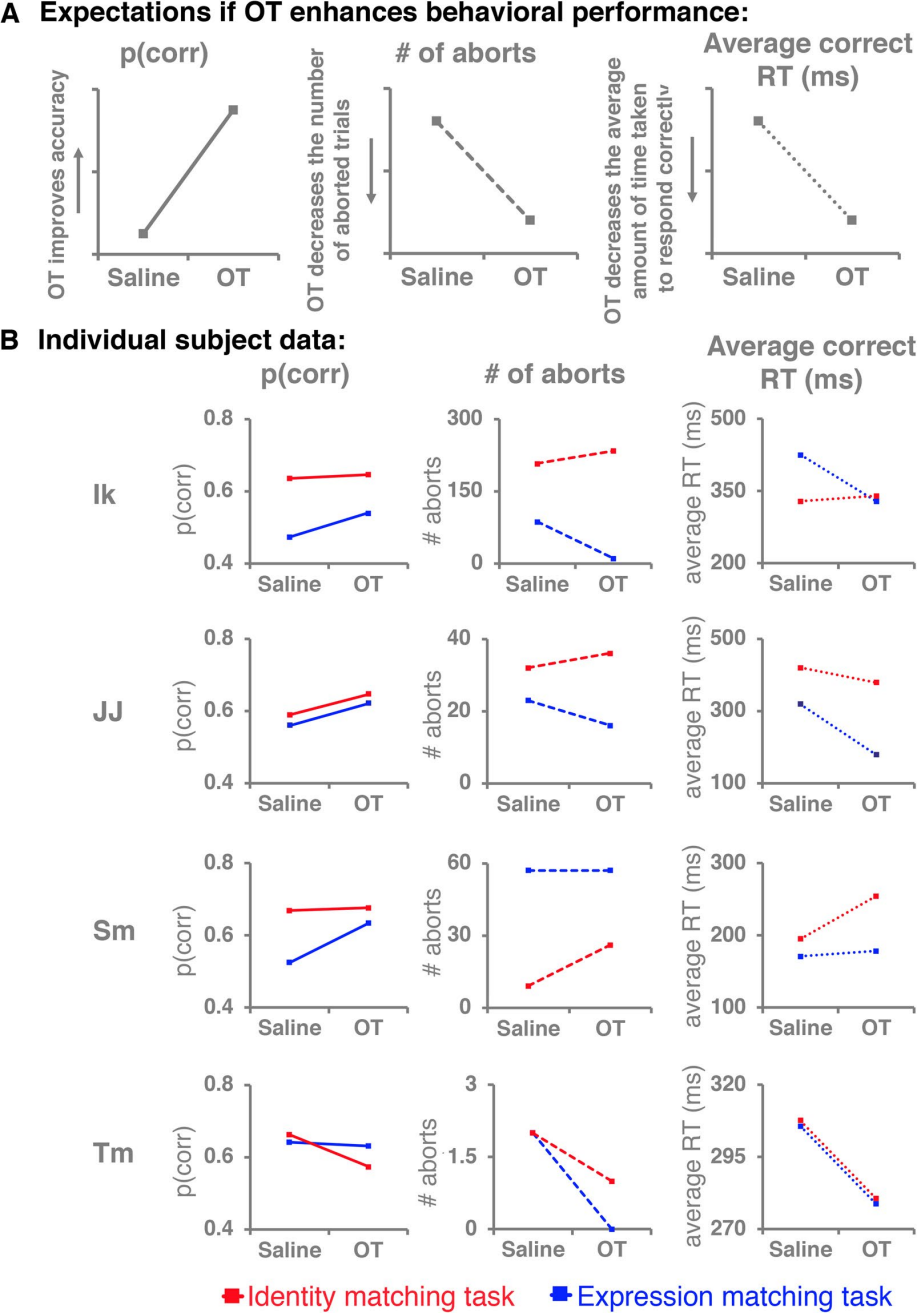
The impact of intranasal OT on individual subject performance. (**A)** The predictations for all three dependment variables if OT improves behavioral performance. **(B)** All three dependent variable plotted for each individual subject. Far left is portion of completed trials that were correct. Middle is the number of aborted trials and on the far right is the average time taken to respond accurately once the choice array was presented. Red lines reflect performance in the identity matching task whereas blue lines reflect performance in the expression matching task.

For each test session, the number of correct responses were computed to the total number of trials completed (excluding aborted trials). We also looked at the number of aborted trials in each test session and the average amount of time the subjects spent looking at the choice array before selecting the correct answer. Note that we subtracted the final 500-ms from every trial because a 500-ms fixation was required to register a reponse. We reasoned that an increase in performance as a result of OT exposure might manifest as increase in accuracy, a reduction in the number of aborted trials, or a decrease in the average reaction time (see Fig. 3A).

When the subjects matched faces based on their identity (see Fig. 3B – red lines), the proportion of correct trials was 0.64 (*SD* = 0.04) when saline was administered before testing and 0.63 (*SD* = 0.05) in the intranasal OT condition, averaging across subjects. The average number of aborted trials increased from 62.5 (*SD* = 97.68) in the saline condition to 74 (*SD* = 107.51) in the OT condition. Finally, the average amount of time the subjects took to respond correctly in both treatment conditions was virtually same (saline-placebo condition *Mean = *313.02-ms, *SD* = 92.43; OT condition *Mean* = 314.51-ms, *SD* = 56.31). These differences in group averages are inconsistent with an enhancement of performance following OT administration.

In contrast, when the subjects looked at the same stimuli but the task at hand was to match faces based on expression (see Fig. 3B – red lines), the average proportion of correct choices increased from 0.55 (*SD* = 0.06) in the saline condition to 0.61 (*SD* = 0.04) in the OT condition. Additionally, the average number of aborts decreased from 42.25 (*SD* = 37.46) in the saline condition to 21 (*SD* = 24.91) in the OT condition, and the average reaction time in correct trials dreeased from 305.39-ms (*SD* = 104.23) in the saline condition to 241.25-ms (*SD* = 75.24) in the OT condition. Overall, these group averages indicate that intranasal OT enhanced performance in the expression matching test. These differences in the group averages are all consistent with enhanced performance following OT administration.

### Experiment 1: The effect of the sample stimulus on identity matching

An important design feature of our experiment is that the stimuli were photographs taken of four different individuals (A, B, C, D), displaying four different expressions (neutral, lip smack, fear grin, open mouth threat). Trials were set up so that each identity and expression appeared as a sample stimulus an equal number of times. This design allowed us to test whether behavior was modulated by the sample’s expression or identity.

**Figure 4:**
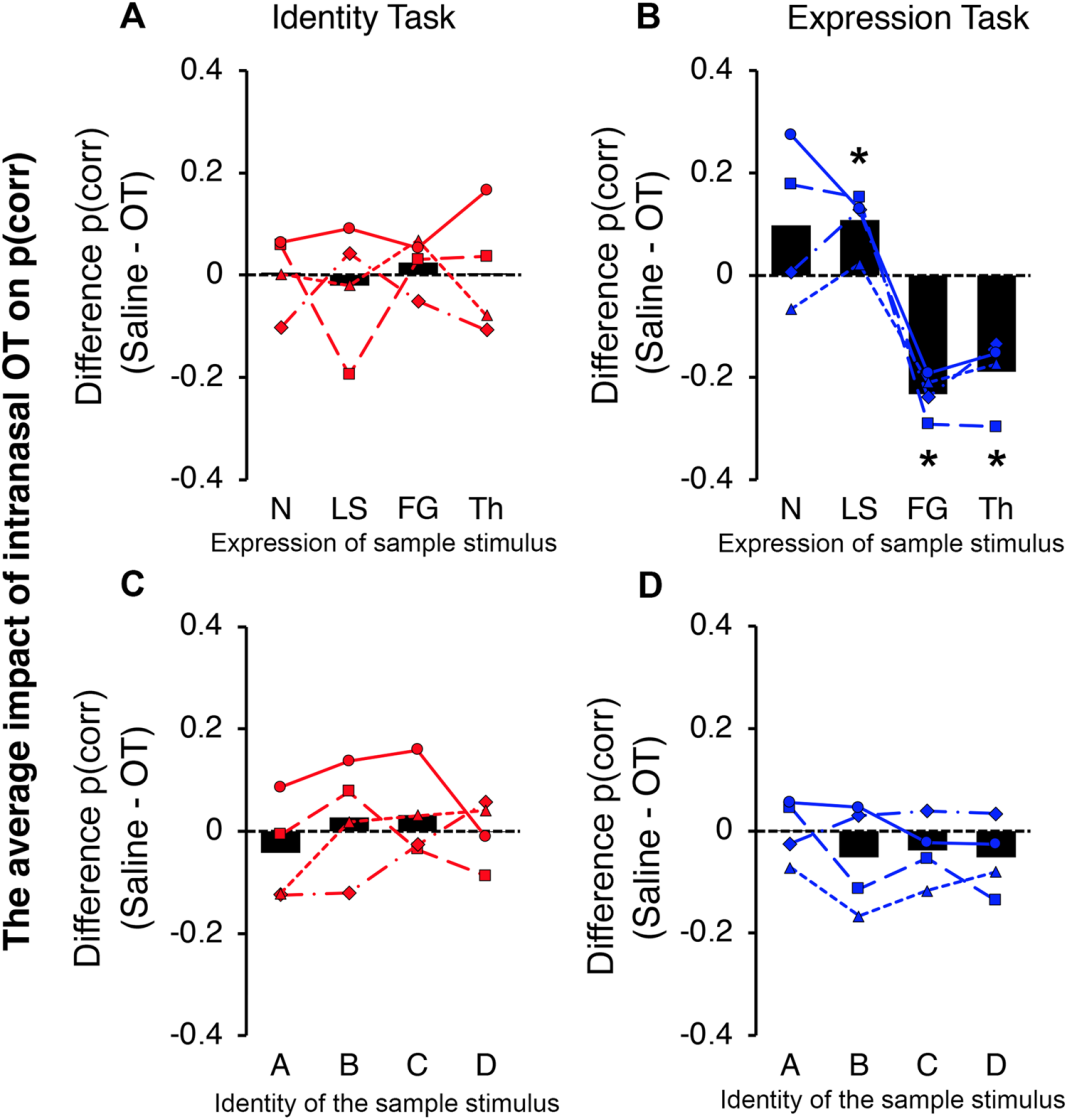
The effect of intranasal OT on accuracy as a function of sample condition. **(A)** The average effect of OT on accuracy in the identity task. The black bars represent the difference in performance following OT administration when averaged across subjects (i.e. [proportion of correct trials in the saline condition] - [proportion of correct trials in the OT condition]). A positive average difference indicates that the subjects responded more accurately in the saline treatment, wheras a negative difference indicates that subject responded more accurately in the OT condition. The superimposed lines represent individual subject results. Here, the results are plotted as a function of the sample’s facial expression and asterisks indicate significant effects of intranasal OT **(B)** The average effect of OT on accuracy in the expression matching task. Same conventions as (A). **(C)** The average effect of OT (saline – OT) on accuracy in the identity matching task as a function of the sample’s identity. Same conventions as (A). **(D)** The average effect of intranasl OT (saline – OT) on accuracy in the expression matching task as a function of the sample’s identity. Same conventions as (A).

To determine whether stimulus expression modulated the effect of intransal OT on the identity matching task, we sorted the test trials by the expression of the sample stimulus (i.e. neutral, submissive lip smack, fear grin and open mouth threat) and subtracted the proportion of correct responses in the intranasal OT condition from the proportion of correct responses in the corresponding saline-placebo condition. Thus, a positive result would indicate that performance was better in the OT condition than in the saline condition. On the other hand, a negative result would mean that intranasal OT delivery improved performance (see Fig. 4A). To analyze these effects at the group level, we performed one sample Bayesian t-tests implemented in R (version 3.6.0) because this method does not rely on large samples (*mu* = 0; equal variance assumed; default priors). We found no evidence that the effect of intranasal OT differed from zero in any of the different trial types (neutral, *M*_*diff*_ = 0.005, *SD* = 0.08, *Bayes Factor* = 2.95, *t*(3) = 0.13, *p* = 0.9; lip smack, *M*_*diff*_ = −0.02, *SD* = 0.12 *Bayes Factor* = 2.82, *t*(3) = −0.33, *p* = 0.8; fear grin, *M*_*diff*_ = 0.02, *SD* = 0.05, *Bayes Factor* = 2.03, *t*(3) = 0.94, *p* = 0.4; threat, *M*_*diff*_ = 0.004, *SD* = 0.12 *Bayes Factor* = 2.96, *t*(3) = 0.06, *p* = 0.9).

We also investigated the impact of the sample’s identity (i.e. A, B, C, D) when subjects matched identity. In this case, the trials were sorted by a manipulation congruent with the subject’s task. For this reason, we thought it was possible that stimulus identity would interact with the impact of intransal OT administration but this was not the case (see Fig. 4A). After we averaged across subject, we found no evidence that the effect of intranasal OT differed from zero in any of the four separate identity conditions (identity A, *M*_*diff*_ = −0.04, *SD* = 0.1 *Bayes Factor* = 2.19, *t*(3) = −0.83, *p* = 0.5; identity B, *M*_*diff*_ = 0.03, *SD* = 0.11, *Bayes Factor* = 2.64, *t*(3) = 0.5, *p* = 0.6; identity C, *M*_*diff*_ = 0.03, *SD* = 0.09 *Bayes Factor* = 2.37, *t*(3) = 0.71, *p* = 0.53; identity D, *M*_*diff*_ < −0.001, *SD* = 0.06 *Bayes Factor* = 2.97, *t*(3) = −0.02, *p* = 0.98). Thus, we found no evidence that the sample stimulus, either its expression or its identity, modulated the effect of OT administration on identity matching. For a post-hoc power analysis based on observed effect size see Table S1.

Independent analyses of the two other dependent variables (i.e the number of aborted trials and time taken to respond correctly) are presented in the Supplementary Material (Figs S1 and S2). In brief, we found no evidence that OT inhalation had a systematic impact on these two measures during identity matching.

### Experiment 1: The effect of the sample stimulus on expression matching

We also examined the influence of the sample face stimulus on expression matching. Organizing the test trials according to the expression of the sample stimulus revealed a striking consistency across subjects (see Fig. 4B). Statistical analysis of the data revealed evidence that the effect of intranasal OT differed from zero in three of the four expression conditions. First, when the sample stimulus expressed a submissive lip smack, the average difference in the proportion of correct responses between the saline and OT conditions was 0.11 (*SD* = 0.06), indicating that OT delivery decreased average accuracy (*Bayes Factor* = 0.301, *t*(3) = 3.69, *p* = 0.03). However, when the sample stimulus had a negatively valenced expression (i.e. a fearful or threatening face), the average difference between the saline and OT conditions was −0.23 (*SD* = 0.04) and −0.19 (*SD* = 0.07) respectively. These differences indicate that when the sample stimulus was a fearful (*Bayes Factor* = 0.034, *t*(3) = −10.685, *p* = 0.002) or threatening face (*Bayes Factor* = 0.152, *t*(3) = −5.18, *p* = 0.01), the average subject accuracy increased when OT was administred, compared to saline. There was no evidence of a significant effect of OT delivery when the sample face was emotionally neutral (*M*_*diff*_ = 0.1, *SD* = 0.16, *Bayes Factor* = 1.62, *t*(3) = 1.25, *p* = 0.3).

It is noteworthy that when the trials were sorted by the identity of the sample stimulus, we found no significant effects of OT administration (identity A, *M*_*diff*_ < 0.001, *SD* = 0.06, *Bayes Factor* = 2.97, *t*(3) = 0.03, *p* = 0.98; identity B, *M*_*diff*_ = −0.05, *SD* = 0.1, *Bayes Factor* = 1.99, *t*(3) = −0.97, *p* = 0.4; identity C, *M*_*diff*_ = −0.04, *SD* = 0.06, *Bayes Factor* = 1.69, *t*(3) = −1.19, *p* = 0.3; identity D, *M*_*diff*_ = −0.05, *SD* = 0.07, *Bayes Factor* = 1.4, *t*(3) = −1.43, *p* = 0.25). For a post-hoc power analysis based on observed effect size see Table S1.

For analysese of the other dependent variables, see the Supplementary Material (Figs S1 and S2). These analyses indicate that OT delivery did not influence the number of aborts or average correct reaction times, when averaged across subject.

### Time course of intranasal OT

Previous research indicates that the influence of OT on our markers of performance would have been delayed in time from the start of the test session^41^. Although we waited for fourty minutes after aerosol treatment before testing, we plotted the proportion of correct responses (relative to the number of both aborted and incorrect trials) as a function of time (see Supplementary Material, Fig. S4) to verify that the time course of OT was not problematic for the interpretation of our results. In these plots, there is no evidence that the time course of OT effects varied systematically across treatment conditions for any of the subjects.

### Experiment 2: catch trials

In Experiment 1, the subjects were required to alternate between the identity and expression matching behaviors a number of times. Thus, its possible that instead of switching between these behaviors, the subjects developed an alternate strategy; the subjects could have learned a matching rule in the first instance (e.g. the correct answer is the matching facial identity), and then learned to reject the matching identity in the subsequent expression task. We note that a rejection strategy would not have promoted accurate performance in the training trials nor the surrounding maintenance trials; thus, it is unlikely that this was the case. Nonetheless, we could not rule it out based on the test data in the first experiment. Therefore, in a second experiment, we reran the saline-placebo condition from Experiment 1 after substituting a subset of test trials with ‘catch trials’. In a catch trial, the distractor did not match the sample at all – not in terms of identity or expression (see Fig. 1). Because there was no relationship between the sample stimulus and the distractor, it could not be used, directly or indirectly, to support performance. We hypothesized that if the subjects were using identity information to complete expression test trials, or expression information to complete identity test trials, then they should fail catch trials in the respective tests and perform at chance.

An additional feature of the catch trials was that the distractor had the same valence as the target stimulus. In regular test trials there had always been one positive face (i.e. a face conveying a neutral expression or a submissive lip smack) and one negative face (i.e. a face conveying a fearful or threatening expression). In contrast, to ensure that responses were not influenced by aversions to faces with negative social value, each catch trial presented subjects with a choice between two negatively valenced faces or two positively valenced faces.

There were 240 “catch trials” during each test phase. These trials were presented randomly throughout the test sessions, surrounded by a larger number of maintenance trials and true test trials. Catch trials were unique to each task; thus, each catch trial was only completed once by each monkey. Since there was no opportunity to repeat a catch trial, it is impossible for the subjects to have learned responses to distinct trials.

In the identity task, we found that all four monkeys performed catch trials significantly above chance (determined using independent binomial tests, two-tailed, numerical chance = 50% of completed catch trials; see Table 1). All four monkeys also performed expression catch trials significantly above chance (determined using two-tailed binomial tests with numerical chance = 50% of completed catch trials; see Table 1). Therefore, the results refute the argument that the subjects were using a rejection strategy. Rather, the results are consistent with the subjects being able to track changes in the task (identity matching and expression matching) when selecting the matching choice stimulus.

**Table 1:** Individual results of catch trials.

Condition	Identity Catch Trials				Expression Catch Trials			
Monkey	Ik	JJ	Sm	Tm	Ik	JJ	Sm	Tm
# completed catch trials	181	239	238	237	235	240	238	239
# correct responses	163	164	177	170	134	166	178	147
p(corr)	0.90	0.69	0.74	0.72	0.57	0.69	0.75	0.62
chance (50%)	91	120	119	119	118	120	119	120
obs. P (2-tailed)	<0.0001	<0.0001	<0.0001	<0.0001	0.0366	<0.0001	<0.0001	0.0005

## Discussion

In this study, we developed a behavioral task to test identity and expression matching performance in rhesus monkeys without changing the visual input. We found that after intranasal OT delivery, average accuracy in the expression matching task numerically improved more so than in the identity matching. A detailed analysis revealed that this improvement in expression matching was driven by enhanced performance in trials where the sample stimulus was expressing fear or aggression (i.e. negatively valenced emotions). The selective influence of OT inhalation on behavioral responses to negatively valenced facial expressions supports the theory that OT effects are tuned to the socio-emotional value of a visual stimulus and that the OT signaling pathway promotes social cognition in a specific way.

Another interesting observation in the results of the expression matching task was that, when the sample stimulus expressed an affiliative lip smack, accuracy systematically decreased in the OT condition. Lip smack facial expressions, which signal appeasement and submission to a rhesus monkey, and are considered to have positive social value, or valence, unlike fearful or aggressive faces which have negative value. The opposing effects of OT depending on the valence of the sample stimulus (see Fig. 4B) were likely driven by the influence of the target – distractor pairings. For example, in Experiment 1, when the sample stimulus and the corresponding target were emotionally neutral or positive (lip smack), the distractor was a face with negative valence. Faces signaling fear or aggression are thought to be aversive to primates because they indicate that something in the environment is potentially harmful or hostile. Actively avoiding looking at negatively valenced faces would have enhanced performance when the sample stimulus was a face with a neutral or submissive expression because the target would have been inherently more pleasant to look at than the distractor. In contrast, when the sample stimulus was expressing fear or aggression, the distractors were positively valenced. Thus, the target faces would have been more adversive to look at, and thereby select, than the distractors, despite the promise of a juice reward. Indeed, the pattern of responses across all four levels of facial expression in the saline-placebo condition (see Supplementary Material, Fig. S3,B) indicates that the subjects actively avoided selecting the fearful and threatening faces, even when they represented the correct answer.

However, after OT delivery, the number of accurate responses in the fearful and threating conditions increased and, likewise, the number of accurate responses in the neutral and lip smack conditions decreased. This interaction was remarkably consistent across all four subjects (see Fig. 4B for individual subject data), demonstrating that the administration of intranasal OT attenuated the spontaneous bias against selecting negatively valenced stimuli that had overridden a trained behavior in the saline-placebo condition. We note that intranasal OT did not have the same impact on subject accuracy in the identity matching task, even though both tests were comprised of the exact same trials, and, thus, the subjects were presented with the same stimulus pairs (i.e. they always had to choose between one positive and one negative facial expression).

The top row Fig. 4A illustrates the effect of intranasal OT on identity matching as a function of the sample’s expression. If intranasal OT had simply dampened a bias against looking at aversive visual stimuli, irrespective of the task at hand, then we should have observed the same pattern of behavior in the identity and expression matching tasks, but this was not the case. Although it is possible that the subjects were looking at and relying on different facial features to discriminate identity and expression, this is outside the scope of the current study because the experimental protocol was not optimized to elicit differences in viewing strategies (e.g., the subjects were given buttons and fixation spots which artificially guided fixations). Nonetheless, because the impact of intranasal OT on behavior towards different facial expressions was limited to the expression matching task, and did not generalize to the identity matching task, our data are inconsistent with the argument that OT in general lowered subject anxiety. Instead, our results indicate that OT specifically altered behavior towards faces signaling different emotions when the task was to match facial expressions. This is an important finding because a common concern in the literature is that previous studies that have investigated the impact of intranasal OT on primate behavior have not been able to rule out general anxiolytic effects.

We also found no evidence that the sample’s identity had a systematic effect on subject accuracy in either task (see Fig. 4C,D; also Fig. S3). Although different unfamiliar conspecifics differ in their approachability, oweing factors such as perceived social dominance and attractiveness, the decision to avoid any particular facial identity most likely varies on an individual subject basis. This might explain why we see less consistency across subjects when the trials were sorted by the identity of the sample stimulus. That said, if intranasal OT upregulated general visual cognition by improving subject attention, memory or motivation, then we should have seen an improvement in subject performance across all four stimulus identities following OT administration. This was not the case (see Fig. 4D). Therefore, by including the identity and expression of the sample stimulus as factors in our design, we can demonstrate the specificity of OT delivery.

At present, it remains unclear how intranasal OT might exert its behavioral effects^10,11^. Recently, the amygdala has been linked to the spontaneous bias monkeys exhibit towards faces when freely viewing displays^42,43^. This indicates that the amygdala is involved at an early stage of information processing, when orienting and directing one’s eye movements towards salient environmental stimuli and those with emotional valence. Since a previous functional imaging study found that the response to facial expressions with negative valence in the monkey amygdala was selectively reduced by the delivery of OT^44,45^, it may be that our results from the OT treatment condition reflect a change in the subject’s viewing behavior stemming from altered amygdala activity^20,29,34,46^.

An alternate view that needs to be considered is that the intranasal delivery method leads to significant increases in the typical levels of OT, effecting a wide range of targets^11^. While our data can not address the composition or physiology of the OT signaling system, they indicate that intranasal OT does not modulate the internal state of a subject in such a way that cognitive tasks are generally easier to respond to. The results of the expression matching task alone reveal that behavior towards different facial expressions was altered by OT delivery (see Fig. 4B), whereas behavior towards different facial identities in the *same* test session was unaffected by OT delivery (see Fig. 4D).

The observation that that it is possible to pharmalogically alter behavioral performance in the expression task, and not the identity task, supports the idea that different neural substrates make unique contributions to face perception. A previous fMRI study found that amygdala lesions had no impact on face responsivity (neutral faces > scrambled faces) or face selectivity (neutral faces > non-face objects), suggesting that while insults to the amygdala might influence judgements about emotional valence or social value, other visual judgements related to face perception may remain intact^44,47^. Although more research is needed to determine whether facial identity and expression are processed by independent neural mechanisms^38–40^, our results indicate that intranasal OT selectively alters the sensitivity of monkeys to facial expressions, possibly via an interaction with motivation and reward circuitry in the primate brain (i.e. the amygdala)^34,48,49^.

Importantly, we caution against the over interpretation of these results given that they are based on a sample size of four^50^. This limitation is unavoidable when using a nonhuman primate model but, nonetheless, the selective impact of OT treatment on expression matching performance was clear in all four subjects serving as a crucial proof of concept for our dual behavioral approach, which can be directly translated into human research^28,51^. In this study, we also delivered OT using a nebulizer because it had been successfully used with head-fixed monkeys in previous research^28,29,41,52^. However, using this delivery method can lead to variability in exact dosage due to daily differences in mask placement and subject cooperation^28,53^. To address this, we counterbalanced the order of treatment and task conditions across subjects but, even so, it is possible that the inconsistent results we observed when subjects matched identity could be attributed to the delivery route and, therefore, our final conclusions must be limited to where we found consistent results, i.e. the bias against negative stimulus valence on expression matching and its reduction by means of OT treatment. Despite these shortcomings, the value of studying nonhuman primate behavior, specifically in rhesus macaques, to our understanding of the OT signaling system cannot be overstated. They provide a highly translatable model system for testing how OT regulates the complex social behaviors which informs its clinical applications.

## Materials and Methods

All procedures were in accordance with the Guide for the Care and Use of Laboratory Animals (49) and were approved by the National Institute of Mental Health Animal Care and Use Committee.

### Subjects

We tested four adult male rhesus monkeys (Ik, JJ, Sm and Tm; *Macaca mulatta*). This sample size was limited to the number of available male monkeys in the laboratory who had not been exposed to OT previously^49^. The monkeys were 14–17 years old at the time of testing (weight varying from 6.85–13.4 kg). They were acquired from the same primate breeding facility in the United States where they had social group histories as well as group housing experience until their transfer to the National Institute of Mental Health (NIMH) for quarantine at the age of approximately 4 years. After that, they were individually caged with auditory and visual contact with other conspecifics in a colony room. All subjects were experienced with passive fixation viewing as well as delayed match-to-sample tasks. Each monkey was surgically implanted with a headpost under sterile conditions using isofluorane anesthesia. After recovery, the monkeys were trained to sit in a plastic restraint chair with their heads fixed in a position directly in front of a computer monitor (resolution = 1280 × 800 pixels).

### Visual stimuli

Four unfamiliar rhesus monkeys (i.e. not housed in the same colony rooms as the subjects) were videotaped under a number of slightly different lighting conditions while props were used to elicit different reactions (such as submission, fear, and aggression). From these video files, 25 frames were saved as still images representing each of the four expressions, neutral, lip smack (submission/appeasement), fear grin, and open mouth threat (aggression), displayed by each of the four male monkeys (herein referred to as model monkey A, B, C and D). In total, there were 400 images in our initial stimulus set. All of these images were scaled down to fit a square canvas (200 × 200 pixels in size) and gray scaled. There was variation in the gaze direction of the faces, although heads were fixed in the frontward facing position. Each face image was individually masked so that external cues (such as primate collars and the background) were removed before the luminance and root-mean-square (RMS) contrast of all the images were adjusted to match the mean luminance and contrast values of the entire stimulus set. Faces were displayed on a medium gray background. Not all images were used during Experiments 1 and 2; a subset of 64 images (16 images per identity − 4 neutral faces, 4 lip smacks, 4 fear grins, 4 open mouth threats) were used for training (identity and expression tasks) and another set of 80 were used for the test trials (20 images per identity - 5 neutral expressions, 5 lip smacks, 5 fear grins, 5 open mouth threats).

### Experimental procedure

In this study, we took advantage of the fact that, at any given time, a face carries both an identity and an expression signal^54^ to design a task where the exact same trials (i.e. the same sample stimulus presented with the same distractors) could be used in both the expression and identity tasks (see Fig. 1). We used different stimuli during training and test phases and designed the delayed match-to-sample task so that the monkeys always had to recognize identity or expression across an image change. More detailed descriptions of all design features are described below.

#### Training sessions

The training/test design was based on a standard methodology for interrogating visual cognition in rhesus monkeys^55–62^. Monkeys were trained to perform a delayed match-to-sample task with basic shapes, and clip art images as well as complex objects and faces. Eye position was monitored using an infrared pupil tracking system (ISCAN, Inc., Woburn, MA, USA) with a 4-ms sampling rate. Stimulus presentation and reward delivery were controlled with Cortex software.

After maintaining an arbitrary performance criterion of 80% over 3 months, each monkey was trained to match facial identity over the course of 8 daily sessions. In each daily session, there were 640 identity training trials (Fig. 1). Each trial began with the presentation of a central fixation spot (0.8 degrees of visual angle). The monkeys were required to fixate this spot for 300-ms to initiate the trial (within a square fixation window of 2 degrees of visual angle in width). A sample stimulus was then presented at the center of the screen. When viewed from approximately 57 cm (the distance between the monitors and the subject’s face), images of the sample faces subtended a visual angle of approximately 5° × 5°. The monkey was required to fixate on the sample stimulus for 500 ms (i.e. anywhere within a square fixation window that was 5.1° of visual angle in width). We note that because the sample stimulus appeared directly behind the fixation spot without any delay, it is likely that the subjects learned to hold fixation throughout the sample phase. Failure to fixate the sample stimulus within a 1500-ms period resulted in a “time out” (i.e. a blank screen was presented for 5-sec) before the same trial repeated.

The sample stimulus on any given trial was drawn from the set of 64 training stimuli. Each of the 64 stimuli served as a sample stimulus in 10 trials throughout a training session. When a monkey successfully completed the sample phase of the trial, it was rewarded with a drop of diluted apple juice before the next phase of the trial began. After a 2-sec delay period, a choice display comprised of two faces was presented. These stimuli were equidistant from the center of the screen, 15.4 degrees of visual angle apart along the horizontal axis of the screen when measured from the center of one image to the center of the other. One of these faces was the target identity, i.e. it was another image of the face viewed in the sample phase. The other face was a distractor identity, i.e. a photograph of another monkey not presented in the sample phase. The sample and the target were never the exact same image as each other and trials were balanced so that each image served an equal number of times as a target and a distractor. The target stimulus was presented on the left and on the right of the screen an equal number of times.

In all of the identity training trials, the expression of the sample and choice stimuli were the same; for example, if the sample stimulus was neutral, the target and distractor were also neutral. The choice display was presented together with two response buttons, one to the left edge of the stimulus presented on the left of the screen and the other on the right edge of the stimulus presented on the right of the screen). These buttons were 0.8 degrees of visual angle in diameter, magenta, and 18.6 degrees of visual angle apart. The task for the monkey when presented with the choice stimuli was to select, by means of an eye movement and a 500-ms fixation, the button corresponding to the target stimulus. The buttons were implemented so that the subjects were not required to fixate on the faces themselves for a long period of time (i.e., 500 ms). This behavior resulted in a large reward (three drops of diluted juice) followed by a 500-ms inter-trial interval (see Fig. 2A). Selecting the distractor or failing to select either stimulus within a 4-sec time window resulted in a 5-sec “time out” period. After the time out, the same trial repeated until the monkey responded correctly.

The expression training trials were identical to the identity training trials except that, in any given trial, the sample and choice stimuli had the same identity and the target/distractor choice stimuli differed in expression, rather than in identity (see Fig. 1). Therefore, the task during expression training sessions was to match the expression of the sample stimulus, not the identity (see Fig. 2B).

To complete an experimental condition (identity or expression matching), monkeys needed to complete 8 training sessions, on 8 consecutive days. On the ninth day, they performed the test session (design and procedure described below; see Fig. 2D,E). Two of the monkeys completed identity training and test sessions before completing expression training and test sessions, while the other two completed expression training and test sessions before completing identity training and test sessions. Each of the two tasks was completed once as part of the saline-placebo treatment condition and once as part of the OT treatment condition (2 tasks × 2 treatments = 4 conditions in total). The order of all four unique conditions was counterbalanced across subjects. In total, it took each monkey 36 days to complete Experiment 1.

#### Test sessions

For each condition, after the eight training sessions were completed, the monkeys completed a single session of test trials (608 test trials in total), broken into two blocks of 304 trials (see Fig. 2D,E). This allowed us to give the monkeys a short break during each test session. Each block began with 14 “maintenance trials”, which were training trials brought into the test session to remind the monkeys of the task at hand. These 14 initial maintenance trials were followed by a cycle of 40 test trials and 10 maintenance trials. There were 240 test trials completed in a block, and 480 in a test session. If the monkey responded incorrectly or aborted a trial, the trial was not repeated.

We used 80 unique stimuli in the test sessions (4 monkey identities × 4 expressions × 5 distinct images). Each stimulus served as a sample on three occasions. Each time a sample stimulus was shown in a test trial, the choice array consisted of two faces: (1) a face that matched the identity but displayed an incongruent expression; and (2) a face that matched the expression but displayed an incongruent identity (see Fig. 1). For example, if the sample stimulus displayed a neutral or lip smack expression, the matching identity displayed either a threat or fear grin expression. Conversely, if the sample stimulus displayed a fear grin or threat, the matching identity displayed either a neutral or lip smack expression (see Fig. 2C). We limited the incongruent expressions in this way because the neutral face and lip smack stimuli were perceptually similar and potentially confusable. This same design was used when matching facial expressions across the delay: sample identities A and B were paired only with choices C and D (and vice versa). The monkeys completed the exact same test trials after identity training as they did after expression training.

Forty minutes before a test session began, we administered OT or a placebo (sterile saline) intranasally to the monkeys using a pediatric nebulizer (PARI Respiratory Equipment, Richmond, VA) with an attached canine anesthesia mask^29,41^ because this fit over the nose and mouth of the subjects without allowing leaks. We note that during this period, and for the duration of a test session, the heads of the subjects were fixed in position via their surgically implanted headposts and they were seated comfortably in the horizontal ‘sphinx’ position^63^. The delay period was implemented to ensure OT was effective^16,41,53^. Although we never administered OT during training sessions, we acclimated the subjects to the entire experiment procedure by treating them with sterile saline using the nebulizer and waiting for 40-minutes before starting the training trials. This acclimation was essential to ensure subject cooperation during the test sessions.

The OT dosage was determined by previous work (24 International Units^29,41,49^ in a 1 ml volume). The method of delivery was chosen as it has previously been shown to increase the concentration of the OT compound in the cerebral spinal fluid of rhesus monkeys^16,41,52^. After treatment and the 40-minute rest period, the task itself took approximately 2 hours to complete during the test sessions. In the Supplementary Material we plotted the performance of each subject, in all four test sessions, as a function of trial order (i.e. time; see Fig. S4). Training sessions were typically longer since incorrect or aborted trials were repeated until the correct response was given.

#### Experiment 2: Catch trials

Our interpretation of the results in Experiment 1 assumes that the monkeys were complying with the task at hand and, therefore, were switching between the identity and expression tasks. To help facilitate this change in task demands, we designed the training and maintenance trials in such a way that the task-irrelevant information was held constant. For instance, in an identity maintenance trial, the target and distractor had the same expression as the sample stimulus. Our goal was to promote the use of the information relevant to the task at hand.

Even so, because the test trials presented the monkeys with a choice between a stimulus with a matching identity and a stimulus with a matching expression, it could be that the monkeys learned a matching rule (e.g. the correct answer is the matching facial identity), and then, instead of switching their focus to facial expression, they might have learned to switch strategies and select the non-matching identity in the expression task. Hence, in this example, performance in the expression task would not reflect expression recognition *per se* but rather the successful rejection of the matching identity. Although it is unlikely that the monkeys adopted this strategy because it would not have supported performance in the preceding training trials nor the surrounding maintenance trials, we could not rule it out based on the data in Experiment 1.

To rule out the possibility that our subjects were using the relationship between the sample stimulus and the distractor to respond to a task, we ran Experiment 2 on each of the four subjects, approximately 3 months (between 84 and 102 days) after the completion of Experiment 1. In this experiment, we reran the saline-placebo condition from Experiment 1 after substituting a subset of the test trials (240 in total) with catch trials. In catch trials the distractor did not match the sample at all – not in terms of identity or expression (see Fig. 1). Because there was no relationship between the sample stimulus and the distractor, it could not be used, directly or indirectly, to support performance. We expected that, if the monkeys were using identity information to complete expression test trials, or expression information to complete identity test trials, then they should fail catch trials in the respective tests and perform at chance.

## Supplementary information

Supplementary Material

Supplementary Dataset 1

Supplementary Dataset 2

## Data Availability

Test data will be made available with the manuscript as Supplementary Information.

## References

[1] Donaldson ZR, Young LJ (2008). Oxytocin, vasopressin, and the neurogenetics of sociality. Science (New York, N.Y.).

[2] Insel TR (2010). The challenge of translation in social neuroscience: a review of oxytocin, vasopressin, and affiliative behavior. Neuron.

[3] McCall C, Singer T (2012). The animal and human neuroendocrinology of social cognition, motivation and behavior. Nature neuroscience.

[4] Modi ME, Young LJ (2012). The oxytocin system in drug discovery for autism: animal models and novel therapeutic strategies. Hormones and behavior.

[5] Parker KJ (2014). Plasma oxytocin concentrations and OXTR polymorphisms predict social impairments in children with and without autism spectrum disorder. Proceedings of the National Academy of Sciences of the United States of America.

[6] Leknes S (2012). Oxytocin enhances pupil dilation and sensitivity to ‘hidden’ emotional expressions. Social Cognitive and Affective Neuroscience.

[7] Ohman A, Flykt A, Esteves F (2001). Emotion drives attention: detecting the snake in the grass. Journal of experimental psychology. General.

[8] Striepens N (2012). Oxytocin facilitates protective responses to aversive social stimuli in males. Proceedings of the National Academy of Sciences.

[9] Norman GJ (2011). Selective influences of oxytocin on the evaluative processing of social stimuli. Journal of psychopharmacology (Oxford, England).

[10] Evans SL, Dal Monte O, Noble P, Averbeck BB (2014). Intranasal oxytocin effects on social cognition: a critique. Brain research.

[11] Leng G, Ludwig M (2016). Intranasal Oxytocin: Myths and Delusions. Biological psychiatry.

[12] Churchland PS, Winkielman P (2012). Modulating social behavior with oxytocin: how does it work? What does it mean?. Hormones and behavior.

[13] de Oliveira DC, Zuardi AW, Graeff FG, Queiroz RH, Crippa JA (2012). Anxiolytic-like effect of oxytocin in the simulated public speaking test. Journal of psychopharmacology (Oxford, England).

[14] Kosfeld M, Heinrichs M, Zak PJ, Fischbacher U, Fehr E (2005). Oxytocin increases trust in humans. Nature.

[15] Zak PJ, Stanton AA, Ahmadi S (2007). Oxytocin Increases Generosity in Humans. PloS one.

[16] Chang SWC, Barter JW, Ebitz RB, Watson KK, Platt ML (2012). Inhaled oxytocin amplifies both vicarious reinforcement and self reinforcement in rhesus macaques (&lt;em&gt;Macaca mulatta&lt;/em&gt;). Proceedings of the National Academy of Sciences.

[17] Bartz JA (2010). Oxytocin selectively improves empathic accuracy. Psychological science.

[18] Thienel M (2016). Oxytocin’s inhibitory effect on food intake is stronger in obese than normal-weight men. International journal of obesity (2005).

[19] Hess L, Votava M, Malek J, Kurzova A, Sliva J (2016). Sedative effects of intranasal oxytocin in rabbits and rhesus monkeys. Physiological research.

[20] Gamer M, Zurowski B, Buchel C (2010). Different amygdala subregions mediate valence-related and attentional effects of oxytocin in humans. Proceedings of the National Academy of Sciences of the United States of America.

[21] Guastella AJ, Mitchell PB, Dadds MR (2008). Oxytocin increases gaze to the eye region of human faces. Biological psychiatry.

[22] Parr LA (2014). Intranasal oxytocin enhances socially-reinforced learning in rhesus monkeys. Frontiers in Behavioral Neuroscience.

[23] Hurlemann R (2010). Oxytocin enhances amygdala-dependent, socially reinforced learning and emotional empathy in humans. The Journal of neuroscience: the official journal of the Society for Neuroscience.

[24] Ebitz RB, Watson KK, Platt ML (2013). Oxytocin blunts social vigilance in the rhesus macaque. Proceedings of the National Academy of Sciences.

[25] Jiang Y, Platt ML (2018). Oxytocin and vasopressin flatten dominance hierarchy and enhance behavioral synchrony in part via anterior cingulate cortex. Scientific reports.

[26] Shamay-Tsoory SG (2009). Intranasal administration of oxytocin increases envy and schadenfreude (gloating). Biological psychiatry.

[27] Neumann ID, Torner L, Wigger A (2000). Brain oxytocin: differential inhibition of neuroendocrine stress responses and anxiety-related behaviour in virgin, pregnant and lactating rats. Neuroscience.

[28] Bauman MD, Murai T, Hogrefe CE, Platt ML (2018). Opportunities and challenges for intranasal oxytocin treatment studies in nonhuman primates. American journal of primatology.

[29] Dal Monte O, Noble PL, Costa VD, Averbeck BB (2014). Oxytocin enhances attention to the eye region in rhesus monkeys. Frontiers in neuroscience.

[30] Averbeck BB, Bobin T, Evans S, Shergill SS (2012). Emotion recognition and oxytocin in patients with schizophrenia. Psychol Med.

[31] Andari E (2010). Promoting social behavior with oxytocin in high-functioning autism spectrum disorders. Proceedings of the National Academy of Sciences of the United States of America.

[32] Parr LA, Modi M, Siebert E, Young LJ (2013). Intranasal oxytocin selectively attenuates rhesus monkeys’ attention to negative facial expressions. Psychoneuroendocrinology.

[33] Prehn K (2013). Effects of intranasal oxytocin on pupil dilation indicate increased salience of socioaffective stimuli. Psychophysiology.

[34] Parr LA, Mitchell T, Hecht E (2018). Intranasal oxytocin in rhesus monkeys alters brain networks that detect social salience and reward. American journal of primatology.

[35] Van IMH, Bakermans-Kranenburg MJ (2012). A sniff of trust: meta-analysis of the effects of intranasal oxytocin administration on face recognition, trust to in-group, and trust to out-group. Psychoneuroendocrinology.

[36] Yue T, Yue C, Liu G, Huang X (2018). Effects of Oxytocin on Facial Expression and Identity Working Memory Are Found in Females but Not Males. Frontiers in neuroscience.

[37] Bruce V, Young A (1986). Understanding face recognition. British journal of psychology (London, England: 1953).

[38] Haxby JV, Hoffman EA, Gobbini MI (2000). The distributed human neural system for face perception. Trends in cognitive sciences.

[39] Grill-Spector K, Weiner KS, Kay K, Gomez J (2017). The Functional Neuroanatomy of Human Face Perception. Annual Review of Vision Science.

[40] Freiwald W, Duchaine B, Yovel G (2016). Face Processing Systems: From Neurons to Real-World Social Perception. Annu Rev Neurosci.

[41] Dal Monte O, Noble PL, Turchi J, Cummins A, Averbeck BB (2014). CSF and blood oxytocin concentration changes following intranasal delivery in macaque. PloS one.

[42] Taubert J, Wardle SG, Flessert M, Leopold DA, Ungerleider LG (2017). Face Pareidolia in the Rhesus Monkey. Current biology: CB.

[43] Taubert J (2018). Amygdala lesions eliminate viewing preferences for faces in rhesus monkeys. Proceedings of the National Academy of Sciences of the United States of America.

[44] Hadj-Bouziane F (2012). Amygdala lesions disrupt modulation of functional MRI activity evoked by facial expression in the monkey inferior temporal cortex. Proceedings of the National Academy of Sciences of the United States of America.

[45] Liu, N., Hadj-Bouziane, F., Moran, R., Ungerleider, L. G. & Ishai, A. Facial Expressions Evoke Differential Neural Coupling in Macaques. *Cerebral cortex (Ne**w York, N.Y.: 1991)*, 10.1093/cercor/bhv345 (2016). 10.1093/cercor/bhv345 PMC6075569 26759479

[46] Dal Monte O, Costa VD, Noble PL, Murray EA, Averbeck BB (2015). Amygdala lesions in rhesus macaques decrease attention to threat. Nature communications.

[47] Hadj-Bouziane F, Bell AH, Knusten TA, Ungerleider LG, Tootell RB (2008). Perception of emotional expressions is independent of face selectivity in monkey inferior temporal cortex. Proceedings of the National Academy of Sciences of the United States of America.

[48] Lefevre A (2017). Oxytocin and Serotonin Brain Mechanisms in the Nonhuman Primate. The Journal of neuroscience: the official journal of the Society for Neuroscience.

[49] Liu N (2015). Oxytocin modulates fMRI responses to facial expression in macaques. Proceedings of the National Academy of Sciences of the United States of America.

[50] Walum H, Waldman ID, Young LJ (2016). Statistical and Methodological Considerations for the Interpretation of Intranasal Oxytocin Studies. Biological psychiatry.

[51] Chang SW, Platt ML (2014). Oxytocin and social cognition in rhesus macaques: implications for understanding and treating human psychopathology. Brain research.

[52] Modi ME, Connor-Stroud F, Landgraf R, Young LJ, Parr LA (2014). Aerosolized oxytocin increases cerebrospinal fluid oxytocin in rhesus macaques. Psychoneuroendocrinology.

[53] Freeman SM (2016). Plasma and CSF oxytocin levels after intranasal and intravenous oxytocin in awake macaques. Psychoneuroendocrinology.

[54] Taubert J, Alais D, Burr D (2016). Different coding strategies for the perception of stable and changeable facial attributes. Scientific reports.

[55] Taubert J, Parr LA (2009). Visual expertise does not predict the composite effect across species: a comparison between spider (Ateles geoffroyi) and rhesus (Macaca mulatta) monkeys. Brain and cognition.

[56] Parr LA, Taubert J (2011). The importance of surface-based cues for face discrimination in non-human primates. Proceedings. Biological sciences/The Royal Society.

[57] Taubert J, Parr LA (2011). Geometric distortions affect face recognition in chimpanzees (Pan troglodytes) and monkeys (Macaca mulatta). Animal cognition.

[58] Taubert J, Aagten-Murphy D, Parr LA (2012). A comparative study of face processing using scrambled faces. Perception.

[59] Parr LA, Taubert J, Little AC, Hancock PJ (2012). The organization of conspecific face space in nonhuman primates. Quarterly journal of experimental psychology (2006).

[60] Taubert J, Qureshi AA, Parr LA (2012). The composite face effect in chimpanzees (Pan troglodytes) and rhesus monkeys (Macaca mulatta). Journal of comparative psychology (Washington, D.C.: 1983).

[61] Weldon KB, Taubert J, Smith CL, Parr LA (2013). How the Thatcher illusion reveals evolutionary differences in the face processing of primates. Animal cognition.

[62] Taubert J, Weldon KB, Parr LA (2017). Robust representations of individual faces in chimpanzees (Pan troglodytes) but not monkeys (Macaca mulatta). Animal cognition.

[63] Vanduffel W (2001). Visual motion processing investigated using contrast agent-enhanced fMRI in awake behaving monkeys. Neuron.

