# Supplementary material for: Intranasal oxytocin selectively modulates the behavior of rhesus monkeys in an expression matching task
Source: Sci Rep. 2019 Oct 23;9:15187. doi: 10.1038/s41598-019-51422-3 (PMC6811679; doi:10.1038/s41598-019-51422-3)
Supplement: Supplementary file 1 — Supplementary Material [file 41598_2019_51422_MOESM1_ESM.pdf]

Supplementary Material

Intranasal oxytocin selectively modulates the behavior of rhesus monkeys in an expression matching task.

Jessica Taubert*[1], Molly Flessert[1], Ning Liu[1,2], and Leslie G. Ungerleider[1]

[1] Section on Neurocircuitry, Laboratory of Brain and Cognition, National Institute of Mental Health (NIMH), National Institutes of Health, Bethesda, MD, United States.
[2] State Key Laboratory of Brain and Cognitive Science, Institute of Biophysics, Chinese Academy of Sciences, Beijing, China.

Supplementary Figure

Fig. S1. The effect of OT delivery on the number of aborts as a function of sample condition.

(A) The effect of OT delivery on the number of aborts when subjects were matching identity. Top panel, the trials were organized by sample expression (Ne, Ls, Fr, Th). There is no evidence that the effect of OT delivery is different from zero in any of these conditions (ne, M_{diff} = -2.75, *SD* = 4.42, *Bayes Factor* = 1.63, *t*(3) = -1.24, *p* = 0.3; ls, M_{diff} = -3.5, *SD* = 3.11, *Bayes Factor* = 0.75, *t*(3) = -2.25, *p* = 0.11; fg, M_{diff} = -2.5, *SD* = 3.41, *Bayes Factor* = 1.37, *t*(3) = -1.46, *p* = 0.24; th, M_{diff} = -3.5, *SD* = 5.8 *Bayes Factor* = 1.67, *t*(3) = -1.21, *p* = 0.31). Bottom panel, the trials were organized by sample identity (A, B, C, D). There is no evidence that the effect of OT delivery is different from zero in any of these conditions (A, M_{diff} = -2.5, *SD* = 4.2, *Bayes Factor* = 1.69, *t*(3) = -1.19, *p* = 0.32; B, M_{diff} = -2.5, *SD* = 2.08, *Bayes Factor* = 0.67, *t*(3) = -2.4, *p* = 0.1; C, M_{diff} = -2.5, *SD* = 3.87, *Bayes Factor* = 1.57, *t*(3) = -1.3, *p* = 0.29; D, M_{diff} = -4.75, *SD* = 4.64, *Bayes Factor* = 0.87, *t*(3) = -2.04, *p* = 0.13). **(B)** The effect of OT delivery on the number of aborts when subjects were matching expression. Top panel, the trials were organized by sample expression (Ne, Ls, Fr, Th). There is no evidence that the effect of OT delivery is different from zero in any of these conditions (ne, M_{diff} = 5.75, *SD* = 10.84, *Bayes Factor* = 1.87, *t*(3) = 1.06, *p* = 0.37; ls, M_{diff} = 7, *SD* = 6.78, *Bayes Factor* = 0.86, *t*(3) = 2.06, *p* = 0.13; fg, M_{diff} = 5.25, *SD* = 9.84, *Bayes Factor* = 1.86, *t*(3) = 1.07, *p* = 0.36; th, M_{diff} = 3.25, *SD* = 9.53 *Bayes Factor* = 2.4, *t*(3) = 0.68, *p* = 0.54). Bottom panel, the trials were organized by sample identity (A, B, C, D). There is no evidence that the effect of OT delivery is different from zero in any of these conditions (A, M_{diff} = 4.5, *SD* = 11.56, *Bayes Factor* = 2.27, *t*(3) = 0.79, *p* = 0.49; B, M_{diff} = 5, *SD* = 8.29, *Bayes Factor* = 1.67, *t*(3) = 1.21, *p* = 0.31; C, M_{diff} = 6.25, *SD* = 10.04, *Bayes Factor* = 1.63, *t*(3) = 1.24, *p* = 0.3; D, M_{diff} = 5.5, *SD* = 7.77, *Bayes Factor* = 1.42, *t*(3) = 1.42, *p* = 0.25).

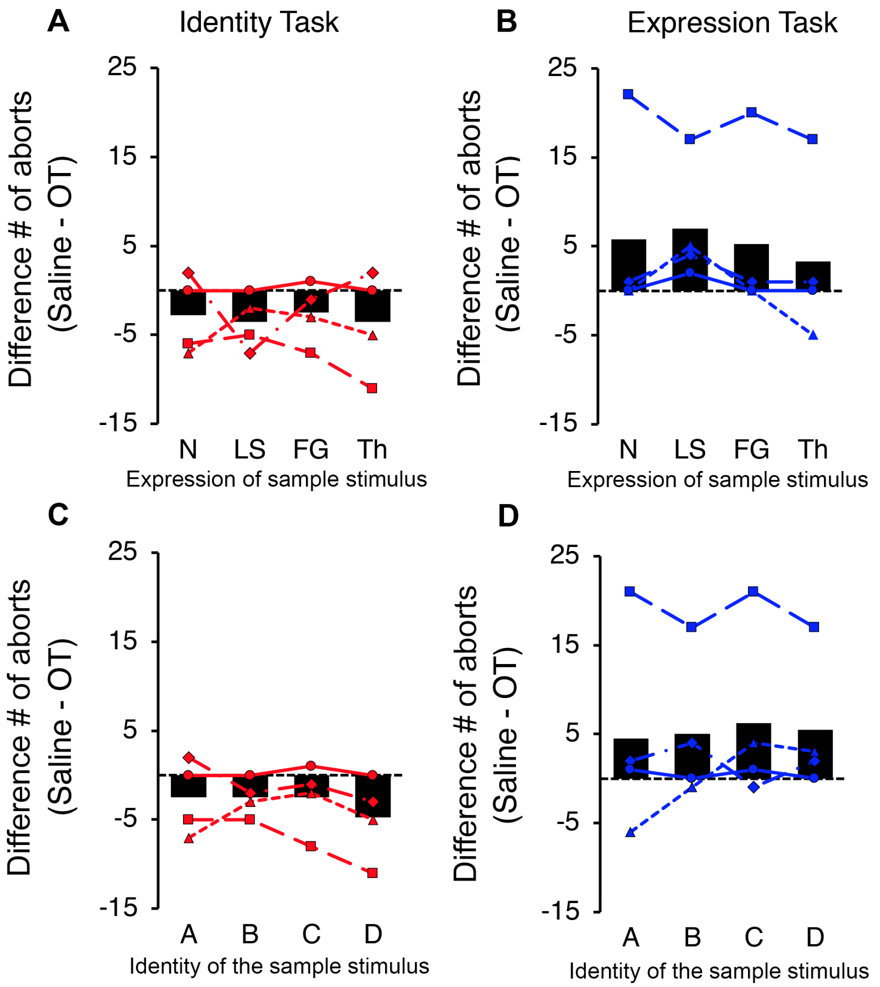

Supplementary Figure

Fig. S2. The effect of OT delivery on the average correct reaction time as a function of sample condition.

(A) The effect of OT delivery on the average correct reaction time (in ms) when subjects were matching identity. The 500 ms required for the selection of the correct choice was subtracted before analysis. Top panel, the trials were organized by sample expression (Ne, Ls, Fr, Th). There is no evidence that the effect of OT delivery is different from zero in any of these conditions (ne, M_{diff} = -6.47, *SD* = 37.6, *Bayes Factor* = 2.81, *t*(3) = -3.4, *p* = 0.75; ls, M_{diff} = 6, *SD* = 63.24, *Bayes Factor* = 2.92, *t*(3) = 0.19, *p* = 0.86; fg, M_{diff} = 12.6, *SD* = 35.2, *Bayes Factor* = 2.36, *t*(3) = 0.72, *p* = 0.53; th, M_{diff} = -20.43, *SD* = 75.58 *Bayes Factor* = 2.6, *t*(3) = -0.54, *p* = 0.63). Bottom panel, the trials were organized by sample identity (A, B, C, D). There is no evidence that the effect of OT delivery is different from zero in any of these conditions (A, M_{diff} = -6.6, *SD* = 21.94, *Bayes Factor* = 2.52, *t*(3) = -0.6, *p* = 0.59; B, M_{diff} = -4.3, *SD* = 31.57, *Bayes Factor* = 2.87, *t*(3) = -0.27, *p* = 0.8; C, M_{diff} = 12.52, *SD* = 44.21, *Bayes Factor* = 2.56, *t*(3) = 0.57, *p* = 0.61; D, M_{diff} = -7.51, *SD* = 87, *Bayes Factor* = 2.93, *t*(3) = -0.17, *p* = 0.87). **(B)** The effect of OT delivery on the average correct reaction time (in ms) when when subjects were matching expression. Top panel, the trials were organized by sample expression (Ne, Ls, Fr, Th). There is no evidence that the effect of OT delivery is different from zero in any of these conditions (ne, M_{diff} = 26.54, *SD* = 60.95, *Bayes Factor* = 2.13, *t*(3) = 0.87, *p* = 0.45; ls, M_{diff} = 52.14, *SD* = 57.68, *Bayes Factor* = 1.04, *t*(3) = 1.81, *p* = 0.17; fg, M_{diff} = 70.62, *SD* = 91.06, *Bayes Factor* = 1.28, *t*(3) = 1.55, *p* = 0.22; th, M_{diff} = 41.84, *SD* = 62.42, *Bayes Factor* = 1.51, *t*(3) = 1.34, *p* = 0.27). Bottom panel, the trials were organized by sample identity (A, B, C, D). There is no evidence that the effect of OT delivery is different from zero in any of these conditions (A, M_{diff} = 46.02, *SD* = 59.95, *Bayes Factor* = 1.29, *t*(3) = 1.54, *p* = 0.22; B, M_{diff} = 14.24, *SD* = 37.93, *Bayes Factor* = 2.31, *t*(3) = 0.75, *p* = 0.51; C, M_{diff} = 67.07, *SD* = 55.53, *Bayes Factor* = 0.66, *t*(3) = 2.42, *p* = 0.09; D, M_{diff} = 46.77, *SD* = 38.98, *Bayes Factor* = 0.67, *t*(3) = 2.4, *p* = 0.1).

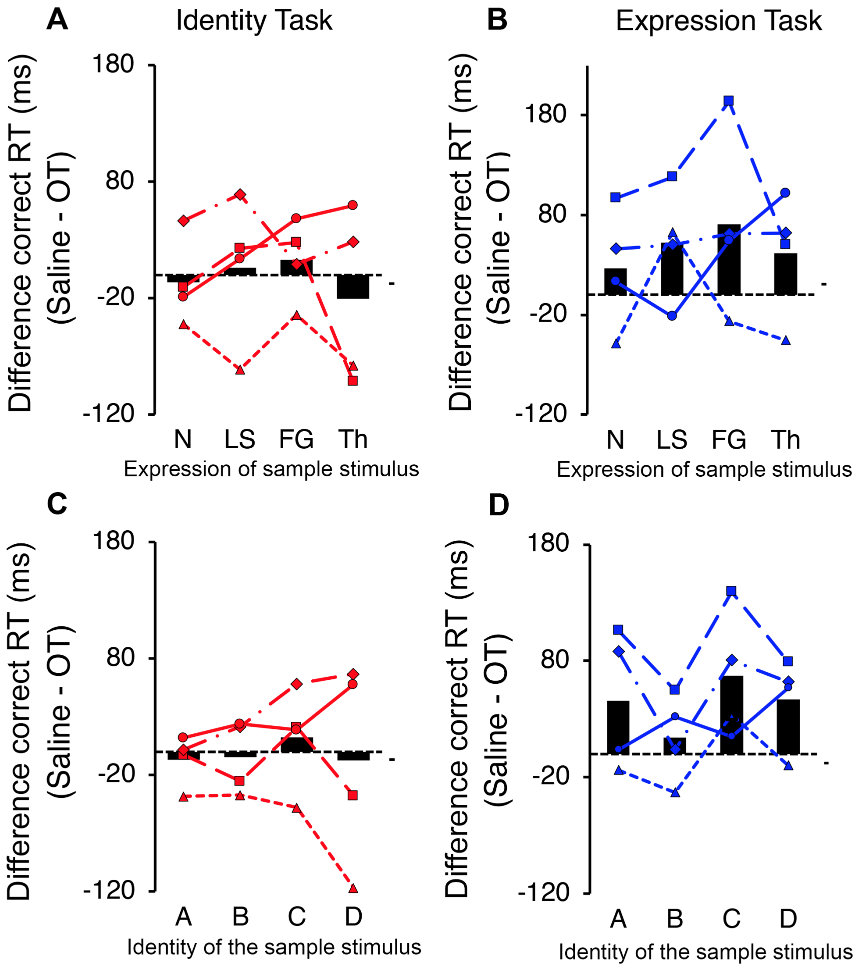

Supplementary Figure

Fig. S3. Results of Experiment 1.

(A) The bar graphs on the left (with red and orange bars) reflect average monkey performance in the identity task as a proportion of the total number of trials completed. Red bars represent average performance when saline was delivered to the monkeys as a placebo before the test session. Orange bars represent performance under the influence of OT. Bar graph on the top depicts performance as a function of stimulus expression, and bar graph at the bottom depicts performance as a function of stimulus identity. Error bars reflect +/- one standard errors of the mean. **(B)** The bar graphs on the right (blue and purple bars) reflect the average proportion of correct responses in the expression task. Same conventions as the graphs on the left, except blue bars represent placebo performance and the purple bars represent performance after OT administration.

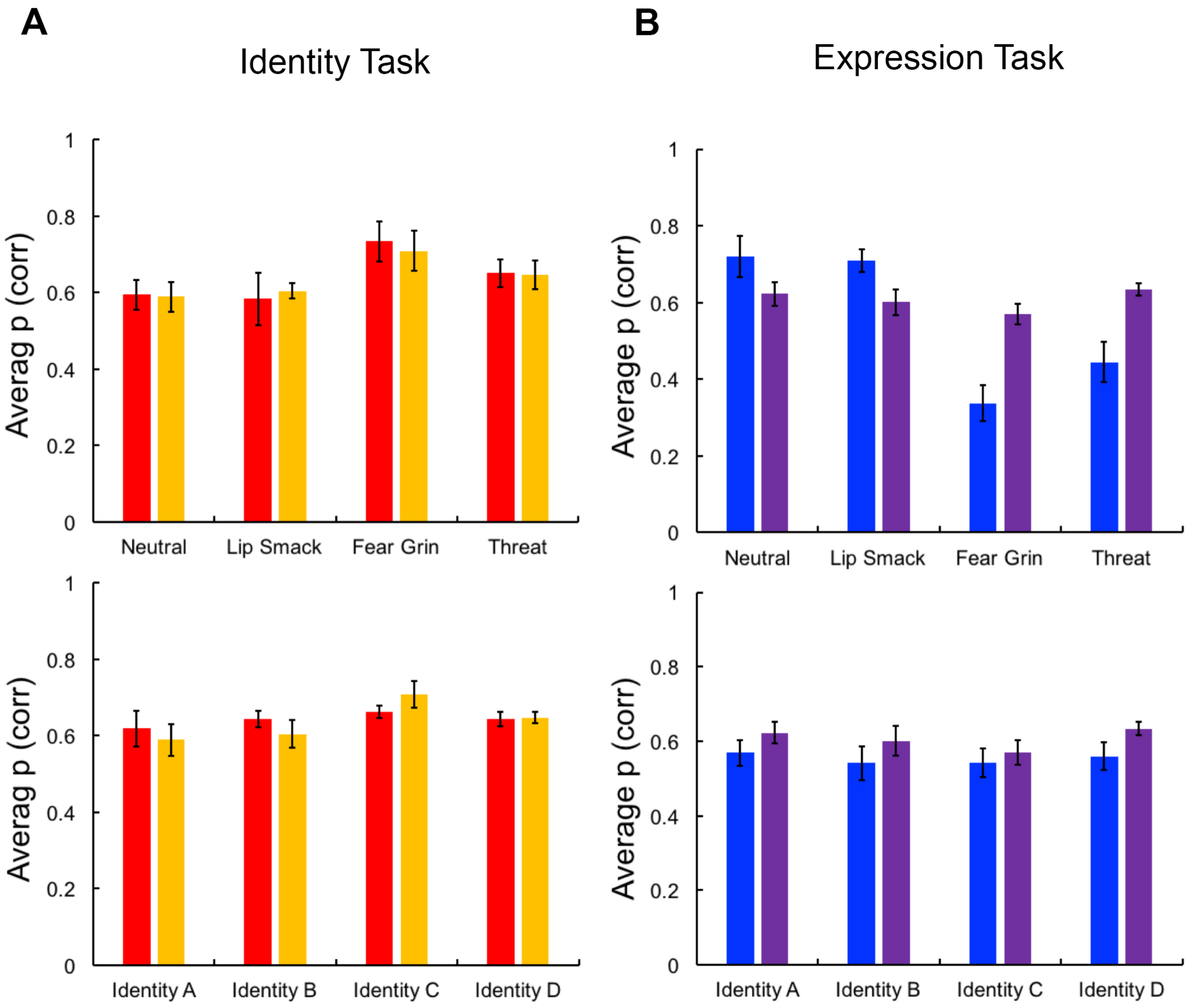

Supplementary Figure

Fig. S4. Performance as a function of trial order.

For each subject (top to bottom: Subjects Ik, JJ, Sm, and Tm), and every experimental condition (separate lines reflect the four conditions in the treatment condition x subject task factorial design), we calculated the average performance using a sliding window (window size = 40 trials, step size = 20 trials). Note that "correct trials" in this case are relative to *all* trials regardless of behavior (i.e. correct, incorrect, and aborted trials). Comparisons between the solid (Saline-placebo conditions) and dotted (OT conditions) lines indicate that the time course of OT (neither its onset nor its decline) was occurred outside the time frame of the test sessions. Dark blue lines reflect responses in the expression matching task while light blue lines reflect responses in the identity matching task.

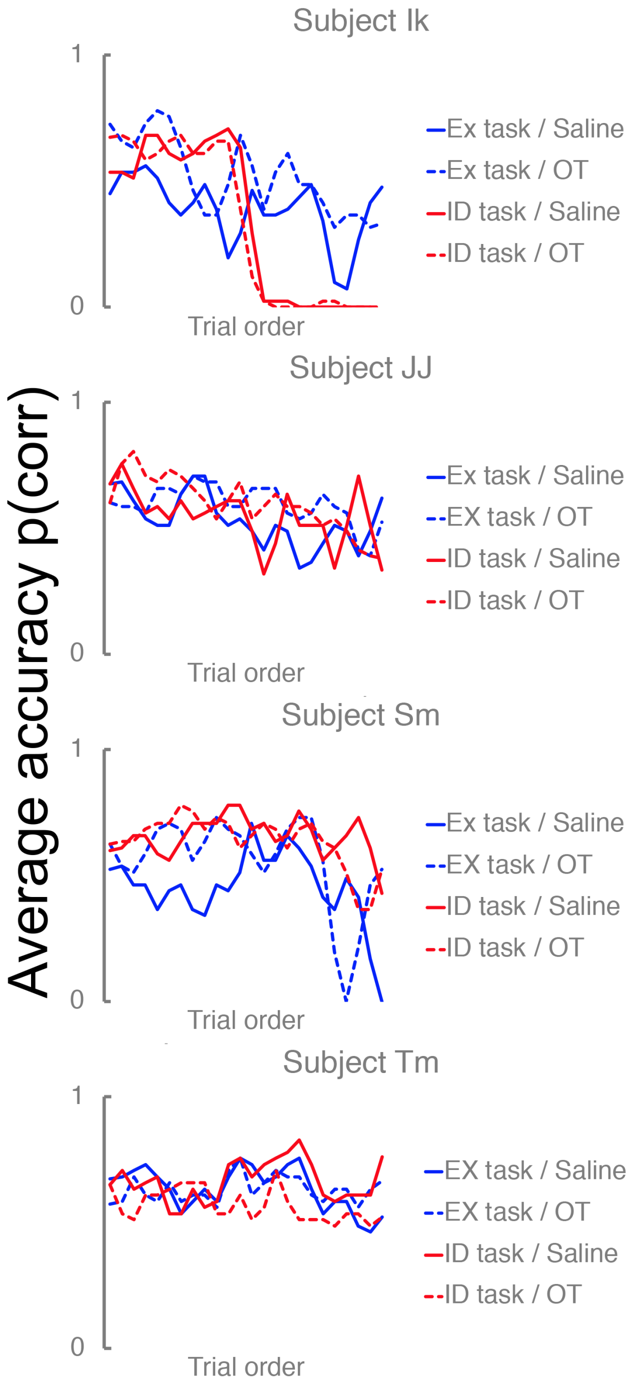

Supplementary Table 1.

Table S1. Post-hoc power analysis of main effects.

Task		Sample condition	One-sample t-test (2-tailed) effective size (d)	Actual Power
**Identity matching**				
	Sample expression	Neutral	0.067	0.051
		Lip smack	0.166	0.057
		Fear grin	0.471	0.104
		Open mouth threat	0.029	0.05
	Sample identity	A	0.416	0.092
		B	0.252	0.065
		C	0.354	0.081
		D	0.008	0.051
**Expression matching**				
	Sample expression	Neutral	0.626	0.146
		Lip smack	2.683	0.931
		Fear grin	5.342	0.999
		Open mouth threat	2.592	0.916
	Sample identity	A	0.016	0.05
		B	0.574	0.131
		C	0.594	0.136
		D	0.715	0.175
